# Circulating bioactive adrenomedullin as a marker of sepsis, septic shock and critical illness

**DOI:** 10.1186/s13054-020-03351-1

**Published:** 2020-11-04

**Authors:** Oscar H. M. Lundberg, Maria Lengquist, Martin Spångfors, Martin Annborn, Deborah Bergmann, Janin Schulte, Helena Levin, Olle Melander, Attila Frigyesi, Hans Friberg

**Affiliations:** 1 Department of Clinical Medicine, Anaesthesiology and Intensive Care, Lund University, 22185 Lund, Sweden; 2 Department of Intensive and Perioperative Care, Skåne University Hospital, 20502 Malmö, Sweden; 3 Department of Anaesthesia and Intensive Care, Kristianstad Hospital, 29133 Kristianstad, Sweden; 4 Department of Anaesthesia and Intensive Care, Helsingborg Hospital, 25437 Helsingborg, Sweden; 5 SphingoTec GmbH, 16761 Henningsdorf, Germany; 6 Department of Infectious diseases, Skåne University Hospital, 20502 Malmö, Sweden; 7 Department of Internal medicine, Skåne University Hospital, 20502 Malmö, Sweden

**Keywords:** Critical illness, Sepsis, Septic shock, Adrenomedullin, Bioactive adrenomedullin, Biomarkers, Cut-off

## Abstract

**Background:**

Biomarkers can be of help to understand critical illness and to identify and stratify sepsis. Adrenomedullin is a vasoactive hormone, with reported prognostic and potentially therapeutic value in sepsis. The primary aim of this study was to investigate the association of circulating bioactive adrenomedullin (bio-ADM) levels at intensive care unit (ICU) admission with mortality in sepsis patients and in a general ICU population. Secondary aims included the association of bio-ADM with organ failure and the ability of bio-ADM to identify sepsis.

**Methods:**

In this retrospective observational study, adult patients admitted to one of four ICUs during 2016 had admission bio-ADM levels analysed. Age-adjusted odds ratios (OR) with 95% CI for log-2 transformed bio-ADM, and Youden’s index derived cut-offs were calculated. The primary outcome was 30-day mortality, and secondary outcomes included the need for organ support and the ability to identify sepsis.

**Results:**

Bio-ADM in 1867 consecutive patients were analysed; 632 patients fulfilled the sepsis-3 criteria of whom 267 had septic shock. The median bio-ADM in the entire ICU population was 40 pg/mL, 74 pg/mL in sepsis patients, 107 pg/mL in septic shock and 29 pg/mL in non-septic patients. The association of elevated bio-ADM and mortality in sepsis patients and the ICU population resulted in ORs of 1.23 (95% CI 1.07–1.41) and 1.22 (95% CI 1.12–1.32), respectively. The association with mortality remained after additional adjustment for lactate in sepsis patients. Elevated bio-ADM was associated with an increased need for dialysis with ORs of 2.28 (95% CI 2.01–2.59) and 1.97 (95% CI 1.64–2.36) for the ICU population and sepsis patients, respectively, and with increased need of vasopressors, OR 1.33 (95% CI 1.23–1.42) (95% CI 1.17–1.50) for both populations. Sepsis was identified with an OR of 1.78 (95% CI 1.64–1.94) for bio-ADM, after additional adjustment for severity of disease. A bio-ADM cut-off of 70 pg/mL differentiated between survivors and non-survivors in sepsis, but a Youden’s index derived threshold of 108 pg/mL performed better.

**Conclusions:**

Admission bio-ADM is associated with 30-day mortality and organ failure in sepsis patients as well as in a general ICU population. Bio-ADM may be a morbidity-independent sepsis biomarker.

## Introduction

### Background

Sepsis is a condition with high mortality and suffering, affecting millions of people yearly across all ages and backgrounds [1].

Since sepsis is a syndrome encompassing a variety of illnesses with multiple pathophysiologies, there is no broadly applicable single efficient treatment pathway.

New methods for stratification and classification of sepsis are warranted in order to better tailor the care of septic patients. The use of biomarkers can potentially help us understand and categorise sepsis into phenotypes [2] and thereby add value to existing risk and severity scoring systems as well as guiding treatment. Further, a better understanding of hormonal systems, which some biomarkers are derived from, can open up for new therapeutical pathways.

### Adrenomedullin

Adrenomedullin (ADM) is a 52-amino acid peptide hormone first discovered in human pheochromocytoma cells [3], but is produced by many different cell types [4]. ADM plays a part in the homeostasis of cardiovascular, endocrine, renal and immunological systems and has a role in the electrolyte balance [3–6]. More specifically, ADM has vasodilatory properties [7, 8] by binding to receptors on both endothelial and smooth muscle cells [9]. Further, ADM is capable of modulating the endothelial barrier, where it has a stabilising effect [9].

### Adrenomedullin in sepsis

Over the last fifteen years, the role of ADM in sepsis has been investigated. Several studies have reported an association of increased levels of ADM and poor outcomes among patients with sepsis and septic shock [10–16]. The role of ADM in patients with a cardiopulmonary disease has also drawn attention [17–24]. These studies have used two assays measuring different fragments from the ADM precursor, mid regional pro adrenomedullin (MR-proADM) [25] and circulating bioactive adrenomedullin (bio-ADM) [12], making results difficult to compare. A cut-off value of 70 pg/mL bio-ADM has been used, which originates from Marino and colleagues [12]. It is not clear how this threshold was chosen, but the authors reported a 100% 28-day survival rate in a minimal subgroup (*n* = 12) where a reduction of bio-ADM levels to below 70 pg/mL was observed.

In animal models of sepsis, however, exogenous ADM has led to improved outcomes[26–28], why ADM has been referred to as a double-edged sword [29]. Further, modulation of the ADM hormonal system using antibodies against a non-ligand binding site of ADM has been suggested a potential therapy in sepsis [30]. This is currently investigated in a phase II clinical trial [31], where septic patients with initial levels of bio-ADM > 70 pg/mL are randomised to receive either the human ADM antibody adrecizumab or placebo [31]. Since ADM levels in non-septic and non-cardiopulmonary critical care patients are poorly investigated, we decided to perform this exploratory study.

### Objectives

The primary aim of this study was to investigate the association of admission bio-ADM with mortality in patients fulfilling the sepsis criteria and in a large mixed general ICU population. Secondary aims were to investigate the association of bio-ADM with organ failure in the ICU, measured as need of circulatory and renal support, and the ability to identify sepsis. Further, we aimed to perform a validation of the proposed cut-off value of 70 pg/mL.

## Methods

### Study design and setting

The present study was a retrospective multicentre observational study of patients consecutively admitted to one of four general (mixed surgical and medical) ICUs in the Skåne Region (Scania county), Sweden, in 2016. The Strengthening the Reporting of Observational Studies in Epidemiology (STROBE) guidelines were followed [32].

### Participants

All adult ICU admissions with valid admission blood samples were included. When direct transfers occurred between the participating ICUs, follow-up data were merged to form cohesive ICU admissions. Transfers from other ICUs were excluded since our aim was to limit our study to primary admissions to intensive care. Information was given to the patient or next of kin, and information letters were sent home to surviving patients 2–6 months after hospital discharge. Patient consent was on an opt-out basis. For deceased patients, consent was presumed.

### Variables

The primary outcome was 30-day mortality in sepsis patients and the general ICU population. Secondary outcomes were: (1) need of cardiovascular support, defined as cardiovascular sequential organ failure assessment (SOFA) score $\ge$ 3, at ICU admission, (2) need for continuous renal replacement therapy (CRRT) during ICU-stay and (3) identification of sepsis at ICU admission.

### Sepsis cohort

The process of identifying the sepsis population, and collection of background data for this cohort, has previously been described in detail [33].

In brief, the sepsis-3 criteria [34] were used to identify patients with sepsis, defined as a SOFA score $\ge$ 2 on ICU admission with a suspicion of infection within 24 h before or 24 h after ICU admission. A suspected infection was defined by blood culture sampling and concomitant administration of oral or intravenous antibiotics (24 h before to 72 h after blood culture), as suggested by the sepsis-3 task force [34].

The predefined exclusion criteria for sepsis admissions were: (1) elective ICU admission after elective surgery, and (2) cardiac arrest within 6 h before or 1 h after ICU admission.

Septic shock was defined as the need of a vasopressor, identified by either a cardiovascular SOFA score $\ge$ 3 or after a medical record review, and a lactate level of $\ge$ 2 mmol/L among those fulfilling sepsis criteria on ICU admission.

### Data sources

Background and survival data were extracted from the patient administrative system for Intensive care units (PASIVA). PASIVA is the portal by which the treating physician and nursing staff submit prospectively collected laboratory and physiological data to the Swedish Intensive Care Registry. PASIVA is synchronised with the Swedish population register, which contains survival data.

Medical records were reviewed retrospectively by trained data collectors to identify sepsis criteria and additional background data [33].

### Bio-ADM measurement

Blood samples, used for the analysis of bio-ADM, were collected on ICU admission and then centrifuged, aliquoted, frozen, and stored in the SWECRIT biobank at Region Scania (BD-47, SC-1922). Samples collected later than 6 h after ICU admission were excluded. If the sampling time was missing, samples were included if the time of freezing was within 6 h. Frozen plasma samples were shipped, and batch analysis of bio-ADM was performed on thawed samples in March 2019 at the laboratory of SphingoTec GmbH (Hennigsdorf, Germany). The assay has previously been described elsewhere [35].

### Study size

The study size was not predetermined but rather a convenience sample. All adult ICU admissions from 2016, with valid admission blood samples and consent, in the SWECRIT biobank constituted our study material.

### Statistics

For all hypothesis tests, we considered *p* values < 0.05 as significant. To assess a difference in the location of two independent variables, we used the Wilcoxon rank-sum test (Mann–Whitney U test). Differences in proportions were assessed using Pearson’s $\chi ^2$ test. Medians were reported with their corresponding interquartile ranges (IQR). The Swedish 2016 calibration of the Simplified Acute Physiology Score III (SAPS3) was used to calculate the estimated 30-day mortality risk (EMR_30-day_) [36, 37]. Multivariable binary logistic regression, adjusted for age, was used to analyse outcomes. The results of the regression analyses are reported as odds ratios (OR) with 95% confidence intervals (CI). The regression models were evaluated with the Hosmer–Lemeshow goodness-of-fit test with ten groups, and models resulting in significant tests were marked [38]. To adjust for severity of disease, SAPS3 was included in the regressions. If a parameter, due to skewness, needed transformation, the base 2 logarithm was used. The difference in Kaplan–Meier curves was evaluated with the log-rank test [39]. Areas under the curve (AUC) were derived from receiver operating characteristic (ROC) curves[40]. Differences in AUCs were tested with the method of DeLong et al. [41]. Youden’s index derived thresholds were reported [42]. Admissions with missing data for any variable were excluded for mean and median calculations. If a variable had missing values, the number of observations available was specified.

**Fig. 1:**
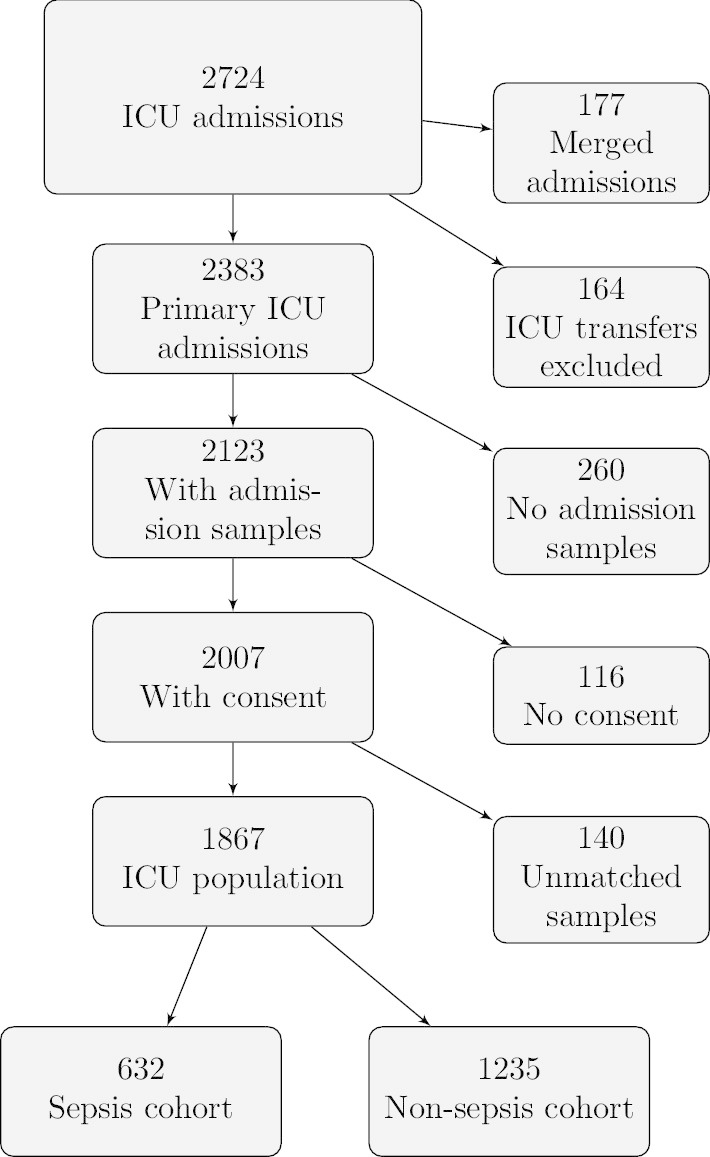
Flow chart of ICU admissions, admission samples and consent. ICU: Intensive care unit

## Results

### Participants

There were 2724 adult admissions in 2016. After merging and exclusion, 1867 admissions with valid samples remained, constituting our study population, shown in Fig. 1. The ICU study population was then divided into a sepsis and a non-sepsis cohort, with 632 and 1235 admissions, respectively.

### Demographics

Patients in the sepsis cohort were generally older and sicker on admission with higher illness severity scores than patients in the non-sepsis cohort, as seen in Table 1. Septic patients were, to a greater extent, admitted from within the hospital, while non-septic patients more often were admitted from the emergency department and directly after surgery. The suspected focus of infection for the sepsis patients is shown in Additional file 1: Table S1. Positive blood cultures with the most common pathogens are displayed in Additional file 1: Fig. S1.

**Table 1:** Demographics and outcomes of the ICU population and a comparison between the sepsis and non-sepsis cohorts

	ICU population	Sepsis cohort	Non-sepsis cohort	*p* value
Number, *n* (% of ICU population)	1867 (100)	632 (33.9)	1235 (66.1)	
Age in years, median (IQR)	67 (54–75)	69 (61–76)	65 (49.5–73)	< 0.001
Female sex, *n* (%)	738 (39.5)	251 (39.7)	487 (39.4)	0.95
*Department of origin*				
Emergency department/out of hospital, *n* (%)	896 (48)	276 (43.7)	620 (50.2)	0.008
Hospital ward, *n* (%)	604 (32.4)	282 (44.6)	322 (26.1)	< 0.001
Intermediate, *n* (%)	50 (2.7)	32 (5.1)	18 (1.5)	< 0.001
Operating room/postoperative ward, *n* (%)	317 (17)	42 (6.6)	275 (22.3)	< 0.001
*Organ dysfunction and illness severity on ICU admission*				
SAPS3 score, median (IQR)	59 (47–71)	66 (57–77)	54 (43–67)	< 0.001
SAPS3 EMR_30-day_, median (IQR)	17.6 (5.2–40.3)	29.9 (14.8–53)	11.1 (3.1–31.9)	< 0.001
SOFA score, median (IQR)	6 (3–9)	7 (5–10)	4 (1–8)	< 0.001
Cardiovascular SOFA score (*n* = 1836), median (IQR)	1 (0–3)	3 (0–4)	1 (0–3)	< 0.001
*Outcomes*				
ICU mortality, *n* (%)	208 (11.1)	86 (13.6)	122 (9.9)	0.019
30-day mortality, *n* (%)	402 (21.5)	174 (27.5)	228 (18.5)	< 0.001
1-year mortality, *n* (%)	622 (33.3)	261 (41.3)	361 (29.2)	< 0.001
ICU length of stay in days, median (IQR)	1.6 (0.8–3.6)	2.5 (1.1–5.5)	1.1 (0.7–2.7)	< 0.001
CRRT use during ICU stay, *n* (%)	169 (9)	96 (15.2)	73 (5.9)	< 0.001
*bio-ADM*				
bio-ADM pg/mL, median (IQR)	40 (21–86)	74 (42–145)	29 (18–56)	< 0.001
bio-ADM> 70 pg/mL, *n* (%)	564 (30.2)	333 (52.7)	231 (18.7)	< 0.001

Data regarding general characteristics, outcomes, organ dysfunction and illness severity are presented. The sepsis cohort was compared to the non-sepsis cohort, and the *p* values refer to that comparison. Proportions (%) are within their subgroups unless otherwise specified. ICU: intensive care unit; IQR: interquartile range; SAPS3: Simplified Acute Physiology Score III; EMR_30-day_: estimated 30-day mortality risk; SOFA: Sequential Organ Failure Assessment; CRRT: continuous renal replacement therapy; bio-ADM: circulating bioactive adrenomedullin

### Outcomes

Mortality rates for the ICU population, sepsis cohort and non-sepsis cohort are shown in Table 1. The sepsis cohort had worse survival data, a greater need for organ support with significantly higher cardiovascular SOFA scores and a higher proportion of CRRT, and a longer ICU stay. A more detailed description of sepsis patients, divided into 30-day survivors and non-survivors, is shown in Table 2. Sepsis patients who did not survive were older and sicker, but with similar pre-existing comorbidities and a similar degree of septic shock on ICU admission, as survivors. Forty-two per cent of sepsis patients fulfilled the septic shock criteria on admission. This subgroup had a 30-day mortality rate of 30.1%, compared to 25.2% in non-shock patients (*p* = 0.15). EMR_30-day_ among septic shock patients was 40.3% (22.5–58.9), while non-shock patients had an EMR_30-day_ of 24.2% (12.2–44.5).

**Table 2:** Demographics and outcomes of the sepsis cohort and comparisons between 30-day non-survivors and survivors

	Sepsis cohort	Non-survivors	Survivors	*p* value
Number, *n* (% of Sepsis cohort)	632 (100)	174 (27.5)	458 (72.5)	
Age in years, median (IQR)	69 (61–76)	73 (66–79)	68 (59–75)	< 0.001
Female sex, *n* (%)	251 (39.7)	61 (35.1)	190 (41.5)	0.17
Body mass index (*n* = 588), median (IQR)	26.6 (22.9–30.7)	26.7 (23.3–31.2)	26.3 (21.8–30.5)	0.11
*Comorbidities*				
None of those listed below, *n* (%)	173 (27.4)	46 (26.4)	127 (27.7)	0.74
Cardiovascular disease, *n* (%)	313 (49.5)	87 (50)	226 (49)	0.95
Respiratory disease, *n* (%)	156 (24.7)	47 (27)	109 (23.8)	0.46
Hepatic disease, *n* (%)	32 (5)	12 (6.9)	20 (4.4)	0.27
Renal disease, *n* (%)	63 (10.0)	18 (10.3)	45 (9.8)	0.96
Cancer, *n* (%)	109 (17.3)	37 (21.3)	72 (15.7)	0.13
Haematological disease, *n* (%)	47 (7.4)	17 (9.8)	30 (6.6)	0.23
Immunosuppression, *n* (%)	126 (19.9)	41 (23.6)	85 (18.6)	0.20
Diabetes, *n* (%)	167 (26.4)	40 (23.0)	127 (27.7)	0.27
Modified Charlson comorbidity index, median (IQR)	1 (0–2)	2 (0–2)	1 (0–2)	0.54
*Department of origin*				
Emergency department/out of hospital, *n* (%)	276 (43.7)	62 (35.6)	214 (46.7)	0.012
Hospital ward, *n* (%)	282 (44.6)	87 (50)	195 (42.6)	0.094
Intermediate, *n* (%)	32 (5.1)	13 (7.5)	19 (4.1)	0.089
Operating room/postoperative ward, *n* (%)	42 (6.6)	12 (6.9)	30 (6.6)	0.88
*Organ dysfunction and illness severity on ICU admission*				
SAPS3 score, median (IQR)	66 (57–77)	76 (66–82)	63 (56–73)	< 0.001
SAPS3 EMR_30-day_, median (IQR)	29.9 (14.8–53)	50.9 (29.9–62.7)	24.2 (13.5–44.5)	< 0.001
SOFA score, median (IQR)	7 (5–10)	9 (6–11)	7 (5–9)	< 0.001
Cardiovascular SOFA score (*n* = 625), median (IQR)	3 (0–4)	3 (1–4)	3 (0–4)	0.037
Septic shock, *n* (%)	267 (42.2)	82 (47.1)	185 (40.4)	0.15
*Outcomes*				
ICU length of stay in days, median (IQR)	2.5 (1.1–5.5)	2.7 (1.2–6.2)	2.4 (1–4.9)	0.16
CRRT use during ICU stay, *n* (%)	96 (15.2)	38 (21.8)	58 (12.7)	0.006
*Biomarkers*				
bio-ADM pg/mL, median (IQR)	74 (42–145)	93 (51–173)	70 (39–131)	< 0.001
bio-ADM > 70 pg/mL, *n* (%)	333 (52.7)	104 (59.8)	229 (50)	0.035
Lactate (*n* = 626) mmol/L, median (IQR)	2.8 (1.5–4.9)	3.3 (1.7–5.7)	2.5 (1.4–4.6)	0.002
CRP (*n* = 600) mg/L, median (IQR)	113 (35–241)	143 (47–238)	102 (32–242)	0.13

Data regarding general characteristics, outcomes, organ dysfunction and illness severity are presented. Non-survivors were compared to survivors, and the *p* values refer to that comparison. Proportions (%) are within their subgroups unless otherwise specified. IQR: interquartile range; SAPS3: Simplified Acute Physiology Score III; EMR_30-day_: estimated 30-day mortality risk; SOFA: Sequential Organ Failure Assessment; ICU: intensive care unit; CRRT: continuous renal replacement therapy; bio-ADM: circulating bioactive adrenomedullin

### Bio-ADM

**Fig. 2:**
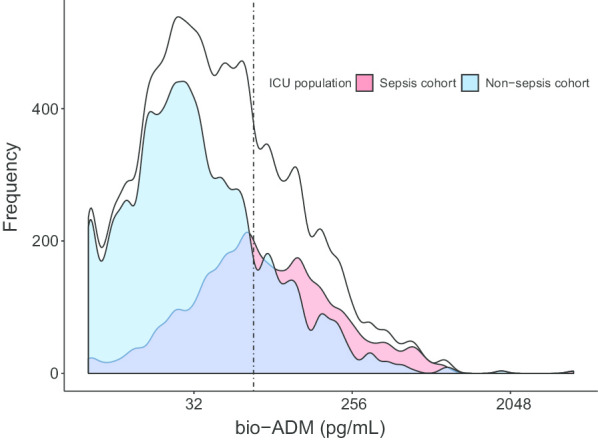
Distribution of bio-ADM in the ICU population, Sepsis cohort and Non-sepsis cohort. X-axis logarithmic with base 2. The dotted line represents a concentration of 70 pg/mL. ICU: intensive care unit; bio-ADM: circulating bioactive adrenomedullin

The range of bio-ADM was 8–4689 pg/mL, and since the distribution was highly skewed, a logarithmic transformation was used, see Fig. 2. The median time from admission to sampling was 25 min (15–40).

### Bio-ADM and mortality

**Fig. 3:**
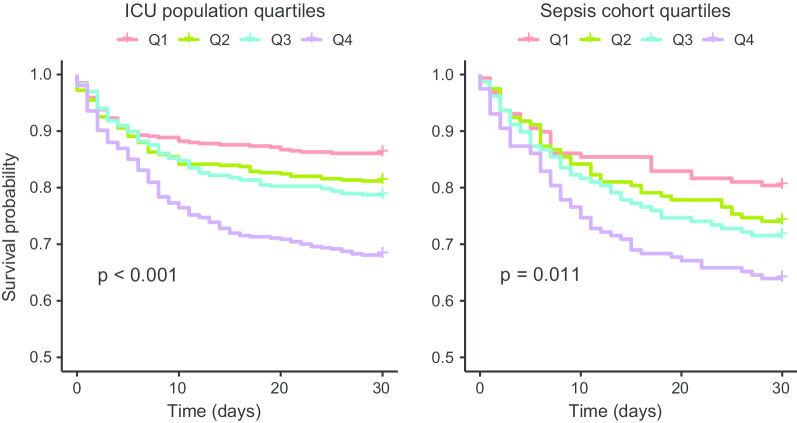
Kaplan–Meier curves for the ICU population and the sepsis cohort according to quartiles of bio-ADM. The range of bio-ADM (pg/mL) in the quartiles in the ICU populations was < 21; 21–40; 40–86; > 86 and in the sepsis cohort < 42; 42–74; 74–145; > 145. The *p* values were derived from the log-rank test. ICU: intensive care unit; bio-ADM: circulating bioactive adrenomedullin; Q1: quartile 1; Q2: quartile 2; Q3: quartile 3 Q4: quartile 4

Dividing patients by quartiles of bio-ADM resulted in significant survival separation in the sepsis cohort as well as in the entire ICU population, as seen in Fig. 3.

Within the sepsis cohort, non-survivors had significantly higher levels of bio-ADM compared to survivors, shown in Table 2.

The associations of bio-ADM in the regression models for 30-day mortality were almost identical in the sepsis cohort and in the entire ICU population, as in Table 3. A doubling of bio-ADM generated a 22–23% increased OR for death.

In the model where admission lactate among septic patients was added as a covariate, bio-ADM was still significantly associated with 30-day mortality with an OR of 1.20 (1.04–1.38). The OR for lactate in the same model was 1.24 (1.06–1.45), *p* = 0.009. When SAPS3 and bio-ADM were applied in the same model for mortality, the association of bio-ADM and mortality was non-significant (data not shown).

The predictive accuracy for bio-ADM and 30-day mortality in the sepsis cohort, presented as AUC, in addition to c-reactive protein (CRP) and lactate are shown in Additional file 1: Table S2.

### Bio-ADM and organ support

The association of bio-ADM with CRRT was strong in the sepsis cohort with OR 1.97 (1.64–2.36) but even stronger in the general ICU population, OR 2.28 (2.01–2.59). The ORs for a cardiovascular SOFA score 3 or 4 were 1.33 for both the septic (1.17–1.50) and the general ICU patients (1.23–1.42), as in Table 3.

### Bio-ADM in sepsis and as a sepsis marker

The median bio-ADM in the sepsis cohort was more than twice as high as the median in the non-sepsis group, as in Table 1. The median bio-ADM in the septic shock subgroup was 107 pg/mL (58–188) compared to 62 pg/mL (35–116) in sepsis patients not presenting with shock ($p< 0.001$). In Table 3, the association of increased bio-ADM levels and the risk of having sepsis and septic shock is presented. The OR of having sepsis in the entire ICU population was 1.78 (1.64–1.94) after adjustment for severity of disease.

In the ICU population, the AUC (95% CI) of bio-ADM to identify sepsis was 0.76 (0.73–0.78), see Additional file 1: Table S2. A Youden’s index derived threshold of 37 pg/mL for detecting sepsis resulted in a sensitivity and specificity of 61% and 80%, respectively.

**Table 3:** Odds ratios for bio-ADM from multivariable binary logistic regression analyses for different outcomes

Outcome	ICU population			Sepsis cohort		
	OR	95% CI	*p* value	OR	95% CI	*p* value
30-day mortality	1.22	1.12–1.32	< 0.001	1.23	1.07–1.41	0.003
30-day mortality^†^	N/A	N/A	N/A	1.20	1.04–1.38	0.010
Cardiovascular SOFA$\ge$ 3	1.33	1.23–1.42	< 0.001	1.33	1.17–1.50	< 0.001
CRRT use during ICU stay	2.28	2.01–2.59	< 0.001	1.97	1.64–2.36	< 0.001
Sepsis	1.91^‡^	1.76–2.08^‡^	< 0.001^‡^^‡^	N/A	N/A	N/A
Sepsis*	1.78^‡^	1.64–1.94^‡^	< 0.001^‡^	N/A	N/A	N/A
Septic shock	1.95	1.76–2.16	< 0.001	1.45	1.28–1.65	< 0.001
Septic shock*	1.78^‡^	1.60–1.98^‡^	< 0.001^‡^	1.35	1.19–1.54	< 0.001

The odds ratio for bio-ADM was calculated on a base 2 logarithmic scale. Age was included as a covariate in all regressions not including simplified acute physiology score III (SAPS3), as this is already an integral part of SAPS3. An additional covariate for the ^†^ model was lactate, and for the * models, the SAPS3 was included. If the Hosmer–Lemeshow test was $p<$ 0.05, the model was marked ^‡^. *ICU: intensive care unit; OR: odds ratio; CI: confidence interval; SOFA: Sequential Organ Failure Assessment; CRRT: continuous renal replacement therapy; N/A: not applicable*

### Bio-ADM cut-offs

**Fig. 4:**
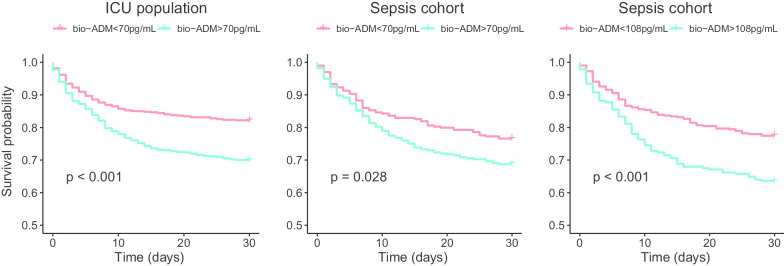
Kaplan–Meier curves for the ICU population and the sepsis cohort according to bio-ADM admission levels above or below 70 pg/mL and according to an Youden’s index derived cut-off of 108 pg/mL for the sepsis cohort. The *p* values were derived from the log-rank test. ICU: intensive care unit; bio-ADM: circulating bioactive adrenomedullin

The cut-off of 70 pg/mL separated the ICU population into high and low bio-ADM, as shown in Table 1. The same information is shown graphically in Fig. 2. The sensitivity for 30-day mortality using a cut-off of 70 pg/mL was 42% with a corresponding specificity of 73% in the ICU population. For the sepsis cohort, the sensitivity and specificity were 60% and 50% for 30-day mortality, respectively. Kaplan–Meier curves and results from log-rank tests for bio-ADM levels above or below 70 pg/mL are displayed in Fig. 4. Youden’s index identified a threshold for survival prediction of 45 pg/mL in the ICU population and 108 pg/mL in the sepsis cohort. A separate Kaplan–Meier curve for the sepsis cohort using the Youden’s index-derived cut-off of 108 pg/mL is shown in Fig. 4. Sensitivity, specificity, positive predictive values, negative predictive values, positive and negative likelihood ratios for all cut-offs are displayed in Additional file 1: Table S2.

## Discussion

In this study, elevated admission bio-ADM levels were associated with increased 30-day mortality in sepsis and in the general ICU population alike. Increased bio-ADM was also associated with cardiovascular failure and need for dialysis. Furthermore, after adjustment of severity of disease, bio-ADM was strongly associated with sepsis.

### Bio-ADM in sepsis

Our sepsis cohort was identified using a structured method where ICU admissions were manually screened for sepsis-3 and septic shock criteria within a narrow time window at ICU admission. Hence, the sepsis diagnosis was not based on discharge diagnose coding, which has been shown to be misleading [33, 43, 44]. We applied predefined exclusion criteria in order to ensure that our sepsis cohort would represent clinically relevant sepsis patients requiring intensive care.

Interestingly, bio-ADM on admission was associated with mortality in sepsis patients and in the general ICU population in a similar fashion. When included in the same regression model for 30-day mortality, lactate and bio-ADM both contributed independently of each other, indicating that bio-ADM carries additional information in sepsis. In line with this, Blet and colleagues, reported added prognostic value of bio-ADM in addition to lactate among septic patients [45].

Bio-ADM has repeatedly been shown to be associated with increased morbidity [15, 16], which also was evident in our study. Sepsis patients were generally sicker and had significantly higher bio-ADM than the general ICU population. Further, patients with septic shock had significantly higher levels of bio-ADM, which is in agreement with previous reports [15, 16, 46].

The association of bio-ADM with sepsis remained after adjusting for severity of disease, implying that elevated levels of bio-ADM on ICU admission makes it more likely that a patient has sepsis.

To our knowledge, there have been no previous reports on the sepsis discriminating properties of bio-ADM in a general ICU population. The ability of bio-ADM to identify sepsis patients was modest with an AUC of 0.76. A Youden’s index derived cut-off of 37 pg/mL generated a sensitivity of 61% and a specificity of 80%, which indicates limited clinical utility of that cut-off.

### Bio-ADM in critical care

The finding that bio-ADM could be broadly applicable to critically ill patients has been reported previously [46]. Lemasle and colleagues studied a large population of patients requiring vasopressor or invasive ventilation for more than 24 h and found an association of bio-ADM with mortality and need for organ support. Their patient population was, however, sicker in comparison with ours. In addition, the bio-ADM samples were not admission samples, which could explain the lower bio-ADM median level in our study (40 pg/mL versus 66 pg/mL).

### Bio-ADM cut-offs

In spite of the questionable rationale of using a cut-off of 70 pg/mL for bio-ADM in sepsis, it has been used in several studies since it was first proposed [12].

In the present study, the 70 pg/mL cut-off managed to separate survivors from non-survivors, but a Youden’s index derived cut-off of 108 pg/mL performed better in sepsis patients, see Fig. 4. Interestingly, Mebazaa et al. reported a similar Youden’s index cut-off of 102 pg/mL from their sepsis cohort in a recent study [15]. For the entire ICU population, the Youden’s index identified the cut-off 45 pg/mL, which is a novel finding for bio-ADM.

### Limitations

There are several limitations to this study.

The study was designed to focus on bio-ADM levels in sepsis patients. All ICU admissions were initially screened for sepsis-3 criteria, and the aim of our data retrieval was primarily to collect detailed data from this cohort. For the remaining ICU population, collection of data was by necessity limited to the PASIVA database, which resulted in different data availability for the sepsis and non-sepsis cohorts. We did, for example, not collect data on comorbidities systematically nor lactate or c-reactive protein levels in the non-sepsis cohort.

We did not have information on the volume status of the patients, nor whether adequate volume resuscitation measures were taken before vasopressor treatment was commenced, a diagnostic criterion for septic shock. However, this limitation is a common feature of studies aiming at identifying septic shock. Initiation of vasopressor therapy in the ICU would usually imply that adequate fluid resuscitation was done, assuming adherence to the Surviving Sepsis Guidelines [47].

We used a strict time frame in which we identified the sepsis and non-sepsis patients and did not investigate the development of sepsis or septic shock beyond that time. Our time constraint may have underestimated the diagnostic value of bio-ADM in sepsis. On the other hand, our method of retrospectively identifying patients fulfilling the sepsis criteria has probably identified patients who were not considered clinically septic by the treating physician.

We were confined to admission samples only, and could not investigate dynamic changes in bio-ADM levels and the impact these may have had on reported outcomes.

The mortality rate in our sepsis and septic shock subgroups was somewhat lower than expected, which could make our results difficult to generalise to patient populations outside of Scandinavia.

## Conclusion

Elevated admission bio-ADM levels correlate with higher 30-day mortality and an increasing need for organ support in both sepsis and non-sepsis ICU patients. Bio-ADM may be an early morbidity-independent marker of sepsis.

## Supplementary information

**Additional file 1.** **Table S1:** Suspected focus of infection and culture findings in the sepsis cohort. **Table S2:** Cutoffs, their corresponding positive and negative predictive values, likelihood ratios and AUCs for the different biomarkers. **Figure S1:** Sepsis patients according to shock status and 30-day survival with one of the eight most common bacteria found in blood cultures are plotted in relation to the suspected focus of infection on ICU admission.

## Data Availability

The datasets generated and analysed during the current study are not publicly available due to limitations in the ethical approval of the study and data management policies of Region Skåne but are available from the corresponding author on request.

## Abbreviations

ADM: Adrenomedullin
AUC: Area under the curve
bio-ADM: Circulating bioactive adrenomedullin
CI: Confidence interval
CRP: c-reactive protein
CRRT: Continuous renal replacement therapy
EMR_30-day_: Estimated 30-day mortality risk
ICU: Intensive care unit
IQR: Interquartile range
MR-proADM: Mid regional pro adrenomedullin
N/A: Not applicable
OR: Odds ratio
ROC: Receiver operating characteristic
SAPS3: Simplified acute physiology score III
PASIVA: Patient administrative system for intensive care units
SOFA: Sequential organ failure assessment
