# Supplementary material for: Circulating bioactive adrenomedullin as a marker of sepsis, septic shock and critical illness
Source: Crit Care. 2020 Nov 4;24:636. doi: 10.1186/s13054-020-03351-1 (PMC7641835; doi:10.1186/s13054-020-03351-1)
Supplement: Supplementary file 1 — Additional file 1. Table S1: Suspected focus of infection and culture findings in the sepsis cohort. Table S2: Cutoffs, their corresponding positive and negative predictive values, likelihood ratios and AUCs for the different biomarkers. Figure S1: Sepsis patients according to shock status and 30-day survival with one of the eight most common bacteria found in blood cultures are plotted in relation to the suspected focus of infection on ICU admission. [file 13054_2020_3351_MOESM1_ESM.pdf]

Additional file 1

Circulating bioactive adrenomedullin as a marker of sepsis, septic shock and critical illness

Table S 1: **Suspected focus of infection and culture findings in the sepsis cohort.** Culture negative patients did not have any positive cultures within the time frame 24 hours before/after ICU admission.

	**Sepsis cohort**
**Suspected focus of infection**	
Respiratory, n (%)	342 (54%)
Gastrointestinal, n (%)	88 (14%)
Cardiovascular, n (%)	6 (1%)
Genitourinary, n (%)	41 (6%)
Musculo-dermato-haematological, n (%)	27 (4%)
Neurological, n (%)	15 (3%)
Unknown, n (%)	113 (18%)
**Sum**	632 (100%)
**Culture findings**	
Positive blood culture, n(%)	139 (22%)
Culture negative	290 (46%)

Table S 2: **Cutoffs, their corresponding positive and negative predictive values, likelihood ratios and AUCs for the different biomarkers.** All cutoffs were Youden's index derived except bio-ADM>70 pg/mL. If data were missing available parameters were specified. *ICU: intensive care unit; AUC: area under the curve; PPV: positive predictive value; NPV: negative predictive value; LR+: positive likelihood ratio; LR-: negative likelihood ratio; bio-ADM: circulating bioactive adrenomedullin; CRP: c-reactive protein*

	**Sensitivity**	**Specificity**	**AUC (95% CI)**	**PPV**	**NPV**	**LR+**	**LR-**
**ICU population**							
**Cutoffs for 30-day mortality**							
Bio-ADM>70 pg/mL	42%	73%	0.61 (0.58-0.64)	30%	82%	1.56	0.79
Bio-ADM>45 pg/mL	59%	58%	0.61 (0.58-0.64)	28%	84%	1.40	0.71
**Cutoff for identification of sepsis**							
Bio-ADM>37 pg/mL	61%	80%	0.76 (0.73-0.78)	51%	86%	2.05	0.33
**Sepsis cohort**							
**Cutoffs for 30 day mortality**							
Bio-ADM>70 pg/mL	60%	50%	0.59 (0.53-0.64)	31%	77%	1.20	0.80
Bio-ADM>108 pg/mL	48%	68%	0.59 (0.53-0.64)	36%	77%	1.51	0.77
CRP>117 mg/L (n=600)	59%	54%	0.54 (0.49-0.59)	32%	78%	1.29	0.75
Lactate>3.1 mmol/L (n=626)	55%	59%	0.58 (0.53-0.63)	34%	77%	1.34	0.76

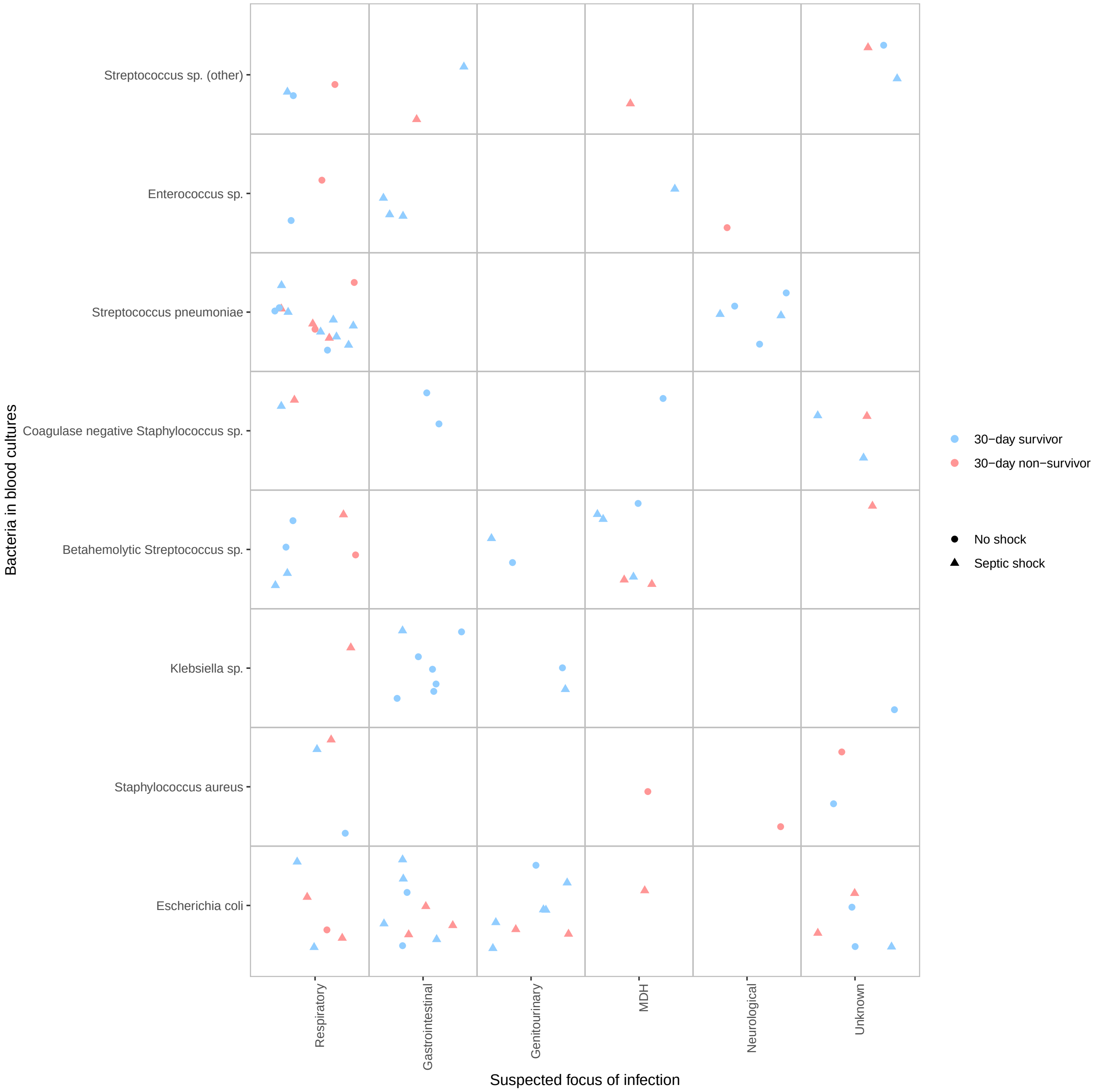

Figure S 1: Sepsis patients according to shock status and 30-day survival with one of the eight most common bacteria found in blood cultures are plotted in relation to the suspected focus of infection on ICU admission. A total of 105 blood culture findings in 98 ICU admissions are included in the figure. Seven admissions had two different bacteria and are thus plotted twice. *MDH, musculo-dermato-haematological*
